# P-2192. The Burden of Respiratory Syncytial Virus Among Young Children in the United States is Not Well-Documented Across Settings: A Systematic Literature Review

**DOI:** 10.1093/ofid/ofaf695.2355

**Published:** 2026-01-11

**Authors:** Mina Suh, Naimisha Movva, Ruvim Izikson, William V La Via, Susan T Pastula, Marina Amaral de Avila Machado, Thomas Shin, Christopher Rizzo

**Affiliations:** Epidstrategies, Mission Viejo, CA; Epidstrategies, Mission Viejo, CA; Sanofi, Bridgewater, New Jersey; Sanofi Pasteur, Swiftwater, Pennsylvania; EpidStrategies, Ann Arbor, Michigan; Sanofi, Bridgewater, New Jersey; Sanofi Pasteur, Swiftwater, Pennsylvania; Sanofi, Bridgewater, New Jersey

## Abstract

**Background:**

Respiratory syncytial virus (RSV) is the leading cause of lower respiratory tract infection (LRTI) and hospitalizations in United States (US) infants and young children. Following ACIP’s recommendation for nirsevimab in infants up to 8 months, we conducted a systematic literature review (SLR) to describe RSV and LRTI epidemiology in US children aged ≥8 months to < 5 years across healthcare settings.

**Figure 1:**
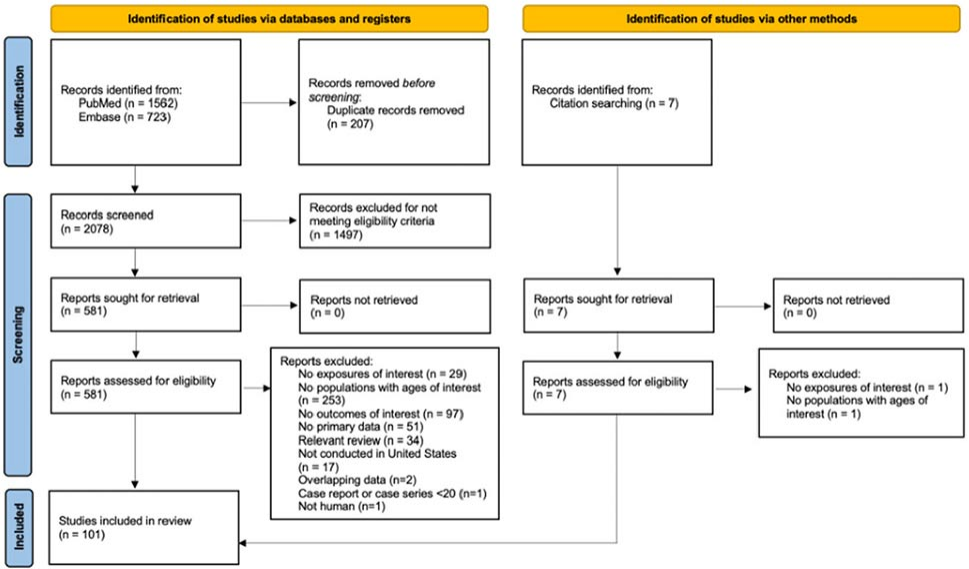
PRISMA Flow Diagram

**Methods:**

This SLR followed PRISMA guidelines and was pre-registered on PROSPERO (#CRD42024599190). Literature published from 2009-2024 were evaluated for outcomes including RSV and LRTI rates in outpatient, urgent care, or emergency department (ED); RSV and LRTI hospitalization rates; and RSV laboratory testing practice and patterns.

**Results:**

This review identified 2085 records; based on the eligibility criteria, 101 studies were included (Figure 1). Of the 101 studies, 34 were prospective cohort, 62 were retrospective cohort, and the remaining 5 were of other designs (1 trial, 1 case-control, 3 cross-sectional surveys). 35 studies provided national-level data; 64 were on various states; 2 were of unknown geographical location. All rate numbers were heterogeneous. 8 studies reported rates in the outpatient or ED settings. In urgent care, no data were available. RSV/LRTI outpatient rates ranged 1.5 to 277.8 per 1000. In the ED, the rates ranged 10 to 84.6 per 1000. RSV hospitalization rates were reported in 26 studies, and the rates were highly variable. RSV laboratory testing patterns were reported in 7 studies with only 1 study providing outpatient data. Though limited, underestimation of RSV is indicated in the outpatient compared to the inpatient setting (testing rates: 69-77% vs. 70-100%, respectively).

**Conclusion:**

This systematic review underscores the significant impact of RSV in US children 8 months through < 5 years of age in all healthcare settings. No data are available for urgent care, and data from outpatient and ED settings remain limited while hospital data are variable. Inconsistent testing and reporting practices may be contributing factors. Given the variable disease burden estimates, additional studies are essential to assess healthcare utilization and impacts in this population.

**Disclosures:**

Mina Suh, MPH, International Health, Moderna: Grant/Research Support|Sanofi: Grant/Research Support|Sobi: Grant/Research Support Naimisha Movva, MPH,Chronic Disease Epidemiology & Regulatory Affairs, Moderna: Grant/Research Support|Sanofi: Grant/Research Support Ruvim Izikson, MD, MPH, Sanofi: Stocks/Bonds (Public Company) William V. La Via, MD, AstraZeneca: Stocks/Bonds (Public Company)|Sanofi: Employee|Sanofi: Stocks/Bonds (Public Company) Susan T. Pastula, MPH, Moderna: Grant/Research Support|Sanofi: Grant/Research Support Marina Amaral de Avila Machado, PhD, Sanofi: Stocks/Bonds (Private Company) Thomas Shin, MA, MPH, Sanofi: Employee of Sanofi|Sanofi: employee and may hold stock or stock options Christopher Rizzo, MD, Sanofi: Employee

